# Supplementary material for: Prevalence of Fatigue and Unrecognized Depression in Patients with Inflammatory Bowel Disease in Remission under Immunosuppressants and Biologicals
Source: J Clin Med. 2021 Sep 11;10(18):4107. doi: 10.3390/jcm10184107 (PMC8471955; doi:10.3390/jcm10184107)
Supplement: Supplementary file 1 [file jcm-10-04107-s001.zip › jcm-1367226-supplementary.pdf]

SUPPLEMENTARY TABLE

Supplementary Table S1. Comparison of the different multivariate regression models.

Model	Variables Included	Adjusted R^2	AIC	*p*-Value
**1**	Medication*, sex, NOD2 variant, resection, TSAT, disease duration◊, Lcn-2	0.141	425.830	0.003
**2**	Medication*, sex, NOD2 variant, TSAT, disease duration◊, EIM	0.123	539.043	0.001
**3**	Medication*, sex, disease type, NOD2 variant, resection, TSAT, disease duration◊◊, EIM, age	0.122	541.894	0.003
**4**	Sex, disease type, NOD2 variant, resection, TSAT, disease duration◊, season	0.111	541.795	0.003
**5**	Medication**, sex, disease type, NOD2 variant, resection, disease duration◊, CRP	0.090	572.426	0.006
**6**	Medication*, sex, ferritin, disease duration◊, BMI, smoking	0.082	397.241	0.035

Model 1 was included in the article after selection based on the adjusted R^2, AIC and p-value.

AIC = Akaike Information Criterion; Lcn-2= lipocalin-2; EIM=extra-intestinal manifestations; TSAT=transferrin saturation; BMI=body mass index.

*Medication categorized: monotherapy with immunosuppressants (IS), adalimumab (ADA), vedolizumab or infliximab (IFX). **Medication categorized: monotherapy with immunosuppressants (IS), monotherapy with a biological or combination therapy. [◊]Disease duration categorized: ≥20 years of IBD compared to <20 years of IBD. [◊◊]Disease duration uncategorized.
